# Serum Adiponectin and Glomerular Filtration Rate in Patients with Type 2 Diabetes

**DOI:** 10.1371/journal.pone.0140631

**Published:** 2015-10-14

**Authors:** Lorena Ortega Moreno, Olga Lamacchia, Massimiliano Copetti, Lucia Salvemini, Concetta De Bonis, Salvatore De Cosmo, Mauro Cignarelli, Vincenzo Trischitta, Claudia Menzaghi

**Affiliations:** 1 Research Unit of Diabetes and Endocrine Diseases, IRCCS Casa Sollievo della Sofferenza, San Giovanni Rotondo, Italy; 2 Unit of Endocrinology, Department of Medical and Surgical Sciences, University of Foggia, Foggia, Italy; 3 Unit of Biostatistics, IRCCS Casa Sollievo della Sofferenza, San Giovanni Rotondo, Italy; 4 Division of Internal Medicine, IRCCS “Casa Sollievo della Sofferenza”, San Giovanni Rotondo, Italy; 5 Department of Experimental Medicine, Sapienza University of Rome, Rome, Italy; UNIFESP Federal University of São Paulo, BRAZIL

## Abstract

High serum adiponectin has been increased in several conditions of kidney disease. Only sparse and conflicting results have been reported in patients with type 2 diabetes (T2D), a subgroup of individuals who are at high risk for renal dysfunction. The aim of this study was to fill up this gap of knowledge by investigating such association in a large sample of Italian diabetic patients. The association between serum adiponectin levels and estimated glomerular filtration rate (eGFR by Chronic Kidney Disease-Epidemiology Collaboration CKD-EPI equation) was investigated in 1,243 patients with T2D from two cross-sectional Italian studies: 878 from San Giovanni Rotondo (SGR) and 365 from Foggia (FG). Serum adiponectin was inversely associated with eGFR in SGR [β (standard error, SE) for 1 standard deviation (SD) of adiponectin = -3.26 (0.64)] and in FG [β(SE)=-5.70(1.28)] sample, as well as in the two studies combined [β(SE)=-3.99(0.59)];(p<0.0001 for all). In this combined analysis, the association was still significant after adjusting for sex, smoking habits, body mass index (BMI), waist circumference, diabetes duration, glycated hemoglobin (HbA1c), albumin creatinine ratio (ACR) and anti-hyperglycemic, anti-hypertensive and anti-dyslipidemic treatments [β (SE)= -2.19 (0.59), p = 0.0001]. A stronger association between each SD adiponectin increment and low eGFR was observed among patients with micro-/macro-albuminuria, as compared to those with normo-albuminuria [adjusted β(SE)=-4.42(1.16) ml/min/1.73m^2^ vs. -1.50 (0.67) ml/min/1.73m^2^, respectively; p for adiponectin-by-albuminuric status = 0.022]. For each adiponectin SD increment, the odds of having eGFR < 60 ml/min/1.73m^2^ increased by 41% (odds ratio, OR = 1.41; 95% confidence interval, CI 1.21–1.64) in SGR sample, 53% (OR = 1.53; 95% CI 1.21–1.94) in FG sample, and 44% (OR = 1.44; 95%CI 1.27–1.64) in the two studies considered together (p<0.0001 for all). In the combined sample, further adjustment for the above mentioned covariates did not change the observed association (OR = 1.36; 95%CI 1.16–1.60; p<0.0001). Our study, so far the largest addressing the relationship between serum adiponectin and GFR in T2D, strongly suggests that the paradoxical inverse association, previously reported in different clinical sets, is also observed in diabetic patients. Further studies are needed to unravel the biology underlying this counterintuitive relationship.

## Introduction

Chronic kidney disease (CKD), mainly indicated by a reduced glomerular filtration rate (GFR), is the leading cause of premature death in patients and type 2 diabetes (T2D) [1–3]. A better understanding of pathogenic mechanisms responsible for GFR decline in T2D is, therefore, urgently needed.

Quite surprisingly, given its beneficial effect on insulin resistance and its anti-inflammatory [4, 5] and anti-oxidative stress [6] properties, circulating adiponectin, a 30 kDa adipokine exclusively secreted by adipocytes in humans [7, 8], is increased in several conditions of kidney disease. Results of the many, often quite small, studies reporting this finding have been properly reviewed [9–11]. Along the same line, a similar counterintuitive association has been reported also in patients with coronary artery disease [12], type 1 diabetes [13–15] and in non diabetic individuals as well [16, 17], but not in individuals with GFR ≥ 60 ml/min/1.73 m^2^ from a population-based Japanese cohort [18]. Despite T2D is strongly characterized by adipocytokines dysregulation [19, 20] and represents one of the most important risk factor for kidney disease [21], no clear data on the relationship between adiponectin and GFR in such condition are available. In details, data on T2D have been mostly obtained in small studies [22–30] whose results are, overall, conflicting, ranging from a direct relationship [25], to no association at all [23, 26, 27, 30], or to an inverse association [22, 24, 28, 29] between adiponectin and kidney function.

To gain further insights into this subject, we analyzed the relationship between serum adiponectin levels and eGFR in more than 1,200 patients with T2D from Central Southern Italy were studied by a cross-sectional design.

## Subjects and Methods

Two independent samples here investigated were recruited from geographically close centers and with identical inclusion and exclusion criteria, thus minimizing possible resulting heterogeneity.

### The San Giovanni Rotondo (SGR) sample

Baseline values of 878 subjects with T2D (defined according to the ADA 2003 criteria) from Gargano (Southern-Centre Italy) were used for this study. They are part of the Gargano Mortality Study, a cohort of consecutively recruited diabetic patients used for prospective investigation on determinants of all-cause mortality in T2D. The general features of this study have been previously described [31–33].

### The Foggia (FG) sample

Baseline values of 365 subjects with T2D (defined according to the ADA 2003 criteria) from Foggia (Southern-Centre Italy) were used for this study. They are part of the Foggia Mortality Study, a cohort of consecutively recruited diabetic patients used for prospective investigation on determinants of all-cause mortality in T2D. The general features of this study have been previously described [31, 32].

### Data Collection and Definitions

Clinical data were obtained from a standardized interview and examination. Body mass index (BMI) was calculated by dividing the weight (in kilograms) by squared height (in meters). Hypertension was defined as a systolic blood pressure was > 130 mmHg or diastolic blood pressure was > 85 mmHg or presence of antihypertensive therapy. Smoking habits, dyslipidemia (as indicated by the presence of lipid-lowering therapy), and anti-hyperglycemic treatment were also recorded at the time of examination. Data regarding medications were confirmed by review of medical records. Individuals who reported smoking cigarettes regularly during the year before the examination were considered current smokers. Diabetes duration was calculated from the current age and the age at diagnosis of diabetes.

In the two studies, blood samples were collected between 8:00 and 9:00 AM after an overnight fast. Serum aliquots were stored at -80°C.

HbA1c was measured, using a HPLC Diamat Analyzer (Bio-Rad, Richmond, CA).

Standardized serum creatinine was measured with the modified kinetic Jaffè reaction (Hitachi 737Autoanalyzer, Tokyo, Japan). The serum creatinine methods had been calibrated to be traceable to an isotope dilution mass spectrometry (IDMS) reference method.

Urinary albumin and creatinine concentrations were determined the same morning of the clinical examination on an early morning first-void sterile urine sample by the Nephelometric method (Nephelometer Analyser; Behring, Germany) and the Jaffe’s reaction-rate method, respectively.

By study design [31, 32], all these variables were assessed at recruitment the same morning of clinical examination.

Estimated GFR was then assessed by CKD-EPI equation [34].

Increased albuminuria was diagnosed if the urinary albumin/creatinine ratio (ACR) was ≥ 2.5 mg/mmol in men and ≥ 3.5 mg/mmol in women.

Presence of urinary tract infections was considered as an exclusion criterion.

### Ethics

The study protocols and the informed consent procedures were approved by the Institutional Ethic Committee of Istituto di Ricovero e Cura a Carattere Scientifico (IRCCS) ‘‘Casa Sollievo della Sofferenza” and of University of Foggia. All participants gave written informed consent.

### Measurement of Circulating Adiponectin Levels

Serum adiponectin concentrations were measured by a commercial ELISA (Alpco, Salem, NH) at the Research Unit of Diabetes and Endocrine Diseases at ‘‘Casa Sollievo della Sofferenza”, as previously described [35]. Inter- and intra-assay coefficients of variation were 7.0 and 6.6%, respectively. The minimum detection limit was 0.019 ng/ml.

### Statistical Methods

Patients’ baseline characteristics are reported as mean ± standard deviation (SD) and percentages for continuous and categorical variables, respectively.

Baseline comparisons between groups were performed using Pearson χ^2^ test for categorical variables, T-test and ANOVA models for normal-distributed continuous variables and Mann-Whitney U test for skewed-distributed continuous variables. To determine normal/no normal distribution Kolgomorov-Smirnorv test was performed.

The association between adiponectin levels and eGFR (i.e. the dependent variable) as a continuous trait was investigated by univariate and multivariable linear regression analysis after logarithm transformation. Covariates were sex, smoking habits, BMI, waist circumference, diabetes duration, HbA1c, ACR, anti-hyperglycemic anti-hypertensive and anti-dyslipidemic treatments. Age was not used as a covariate because it is already included in the CKD-EPI formula and also because of its collinearity with disease duration.

Results were reported as linear model coefficients along with their standard errors [β (SE)]. The association between adiponectin levels and eGFR as a dichotomous trait (i.e. ≥ or < 60 ml/min/1.73m^2^) was tested by logistic regression analysis. Results were reported as ORs, along with their 95% CI.

Random effect meta-analysis was performed in an individual patient data meta-analysis fashion [36] after checking for heterogeneity (i.e. the presence of a significant exposure-by-sample interaction).

A p-value < 0.05 was considered as significant. All analyses were performed using SPSS v.15 (SPSS, Chicago IL) and SAS Release 9.1.3 (SAS Institute, Cary, NC, USA).

## Results

Patients’ clinical features in both samples are shown in Table 1.

**Table 1:** Clinical characteristics of patients from SGR and FG studies.

	SGR (N = 878)	FG (N = 365)
Sex (males %)	456 (51.9)	201 (55.1)
Age (yrs)	61.8±9.6	63.3±11.2
Smokers (%)	128 (14.6)	68 (18.6)
BMI (kg/m^2^)	30.9±5.6	29.7±5.8
Waist circumference (cm)	102.2±13.5	105.3±14.4
Diabetes duration (yrs)	10.7±8.9	13.4±10.2
HbA1c (%)	8.7±2.0	9.0±2.1
Anti-hyperglycemic treatment (%)	733 (83.5)	323 (88.5)
- Oral agents	368 (41.9)	176 (48.2)
- Insulin w/wo oral agents (%)	365 (41.6)	147 (40.3)
ACE/RAS treatment (%)	360 (49.7)	230 (63.0)
Statins treatment (%)	263 (30.0)	135 (37.0)
Micro-/macro-albuminuria (%)	248 (29.8)	155 (43.1)
eGFR (ml/min/1.73m^2^)	73.6±19.1	80.9±25.1
Adiponectin (μg/ml)	6.0 ± 3.6	5.8±3.6

Continuous variables were reported as mean ± SD whereas categorical variables were reported as total frequency and percentages. SGR: San Giovanni Rotondo; FG: Foggia; BMI: Body Mass Index; HbA1c: glycated hemoglobin;, ACE/RAS: angiotensin-converting enzyme/renin-angiotensin system; eGFR: estimated glomerular filtration rate.

The two samples were quite different in terms of most clinical variables (p<0.05) but, sex, anti-hyperglycemic treatment and adiponectin levels.

When eGFR was analyzed as a continuous trait, an inverse association between serum adiponectin and eGFR was obtained: β (SE) for each SD increase of adiponectin level = -3.26 (0.64) ml/min/1.73m^2^ in SGR study; -5.70 (1.28) ml/min/1.73m^2^ in FG study and -3.99 (0.59) ml/min/1.73m^2^ in the two studies combined; with a p value less than 0.0001 for all of them (Table 2).

**Table 2:** Association between serum adiponectin levels and eGFR, considered as a continuous trait.

	SGR (N = 878)		FG sample (N = 365)		Pooled data meta-analysis (N = 1,243)*	
	β (SE)	P	β (SE)	P	β (SE)	P
**Model 1**	-3.26 (0.64)	<0.0001	-5.70 (1.28)	<0.0001	-3.99 (0.59)	<0.0001
**Model 2**	-1.22 (0.65)	0.037	-3.91 (1.21)	0.003	-2.19 (0.59)	0.0001

SGR: San Giovanni Rotondo; FG: Foggia. p value obtained from lnGFR.

The β linear coefficients represent the change in eGFR level (ml/min/1.73m^2^) for 1.SD increase in adiponectin. SE: standard error.

Model 1: unadjusted.

Model 2: adjusted by sex, smoking habits, BMI, waist circumference, diabetes duration, HbA1c, ACR and anti-hyperglycemic, anti-hypertensive and anti-dyslipidemic treatments.

*Since the effect in SGR tended to be different than that in FG sample (p for β values heterogeneity being = 0.15), individual data meta-analysis was carried out in a conservative fashion by using random effects.

Pooled analysis were adjusted by study sample.

These associations were still significant after adjusting for several variables we were able to account for, all possibly influencing renal function: sex, smoking habits, BMI, waist circumference, diabetes duration, HbA1c, ACR and anti-diabetic, anti-hypertensive and anti-dyslipidemic therapies (Table 2).

In the combined analysis, the association between serum adiponectin and eGFR was clearly different in patients with normo-albuminuria as compared to those with micro-/macro-albuminuria [adjusted β (SE) for each SD increase of adiponectin level = -1.50 (0.67) and -4.42 (1.16) ml/min/1.73m^2^, respectively, p = 0.002 for both; p for adiponectin-by-albuminuric status interaction = 0.022]. In contrast, no difference in the association between adiponectin and eGFR was observed in patients with micro- as compared to those with macro-albuminuria (p for adiponectin-by-albuminuric status interaction = 0.30) as well as in men as compared to women (p for adiponectin-by-sex interaction = 0.74).

When eGFR was analyzed as a dichotomous trait of clinical significance (i.e. eGFR < or ≥ 60 ml/min/1.73m^2^), adiponectin levels were higher in patients with lower eGFR in both SGR (n = 175; 7.1±4.1 vs. n = 703; 5.7±3.4) and FG (n = 73; 7.1±3.2 vs. n = 292; 5.4±3.6), as well as in the two studies combined (n = 248; 7.1±3.9 vs. n = 995; 5.6±3.4). For each SD increment in adiponectin levels, the odds of eGFR < 60 ml/min/1.73m^2^ was significantly increased in SGR, FG and in the two studies combined (Table 3).

**Table 3:** Association between serum adiponectin levels and low GFR (< 60ml/min/1.73m^2^).

	SGR sample (N = 878)		FG sample (N = 365)		Pooled data meta-analysis (N = 1,243)*	
	OR (95% CI)	p	OR (95% CI)	p	OR (95% CI)	p
**Model 1**	1.41 (1.21–1.64)	<0.0001	1.53 (1.21–1.94)	<0.0001	1.44 (1.27–1.64)	<0.0001
**Model 2**	1.31 (1.08–1.60)	0.007	1.53 (1.14–2.04)	0.004	1.36 (1.16–1.60)	<0.0001

SGR: San Giovanni Rotondo; FG: Foggia;

OR (95% CI) are given for 1 SD increase of adiponectin levels.

Model 1: unadjusted.

Model 2: adjusted by sex, smoking habits, BMI, waist circumference, diabetes duration, HbA1c, ACR and anti-hyperglycemic, anti-hypertensive and anti-dyslipidemic treatments.

*Since the effect in SGR was not different than that in FG sample (p for OR values heterogeneity being = 0.56), individual data meta-analysis was carried out by using fixed effects.

Pooled analysis were adjusted by study sample.

Also in this case such associations remained significant after adjusting for the several covariates mentioned above (Table 3).

## Discussion

This study, comprising more than 1,200 Italian patients with T2D, shows an inverse relationship between adiponectin levels and GFR. Such counterintuitive association is independent of many confounders we were able to account for.

Previous studies on the relationship between adiponectin and kidney function in T2D have been thus far contradictory [22–30], with some reporting a direct relationship in men (i.e. toward the opposite direction we here report) [25], some reporting no association at all [23, 26, 27, 30], and finally some others describing an inverse association [22, 24, 28, 29], like the one we observed. Unfortunately, most of these studies were carried out in small samples, which definitively lacked the power for drawing firm conclusions [24, 28, 29] or in non-European populations [22], thus leaving unaddressed the association between adiponectin and kidney function among European patients with T2D. So, our study, thanks to the large number of individuals who were investigated, says a strong word on this subject, definitively indicating that a paradoxical association between serum adiponectin and kidney function is observable also among patients with T2D of European origin.

It is of note that such association was similar in men and women, thus making difficult to reconcile our data with those reported in a previous study, carried out only in men and reporting an association toward the opposite direction we here observed [25].

By its intrinsic nature, our observational study does not allow to draw firm conclusions about the possible mechanism(s) underlying this paradoxical association. We can only hypothesize, that in individuals with kidney dysfunction, increased levels of circulating adiponectin, rather than being only a mere effect of decreased renal excretion, represent a tentative homeostatic mechanism aimed at counteracting, through anti-inflammatory and anti-oxidative stress mechanisms [4–6], renal damage [37, 38]. Animal studies have in fact reported that adiponectin retards the progression of diabetic nephropathy [39, 40]. Such a scenario might be also exacerbated by a reduced response to adiponectin biological effects, as suggested by the recent observations of a post receptor adiponectin resistance, paralleled by increased adiponectin and adiponectin receptor 1 gene expression, in peripheral tissues from humans with severe kidney dysfunction [41, 42].

An additional result of our study is that the negative correlation between adiponectin and GFR levels is more evident in patients with micro-/macro-albuminuria as compared with those with normo-albuminuria. How micro-/macro-albuminuria exacerbates the negative correlation between serum adiponectin and GFR is not known. Overall, the stronger association observed in the presence of an additional condition of kidney dysfunction (i.e. increased albuminuria), reinforces the hypothesis of a tentative, protective role of adiponectin as a mechanism underlying its inverse counterintuitive relationship with GFR.

Strengths of our study are the overall sample size, consisting of a total of 1,243 diabetic patients all from the same geographical region and the fact that all samples were handled identically, with measurement of serum adiponectin being centralized.

Conversely, the lack of information on specific classes of anti-hyperglycemic drugs has to be recognized as a limitation.

Despite the two study samples have been recruited from a geographically homogeneous region and with identical inclusion/exclusion criteria, some differences in baseline clinical features emerged. Being aware that such differences might have played a role in the association between adiponectin and kidney function, we were conservative enough to use random-effect in the pooled meta-analysis, thus taking into account possible across samples heterogeneity.

In addition, it is not known whether our present finding is generalizable to other populations of European ancestry with different environmental and/or genetic backgrounds, both factors known to modulate serum adiponectin concentrations [35, 43].

In conclusion, our data clearly show, that there is an inverse and independent association between serum adiponectin and GFR among Italian patients with T2D. Further studies are warranted to examine the exact mechanisms underlying this counterintuitive relationship and to explore the potential role of adiponectin as a tool for improving prediction, prevention and treatment strategies aimed at reducing the burden of kidney function loss in such high risk individuals.
